# Eleven-year patency of ligamentum teres hepatis graft for portal vein and superior mesenteric vein reconstruction following pancreaticoduodenectomy: A case report

**DOI:** 10.1097/MD.0000000000047133

**Published:** 2026-01-09

**Authors:** Zhiwei Liu, Qiang Wei, Wentao Zhu, Xutao Lin, Qiangpu Chen

**Affiliations:** a Department of Hepatobiliary Surgery, Binzhou Medical University Hospital, BinZhou, Shandong Province, China.

**Keywords:** ligamentum teres, neuroendocrine tumors, pancreatoduodenectomy, portal vein, superior mesenteric vein, vascular grafting

## Abstract

**Rationale:**

Pancreaticoduodenectomy combined with portal vein and/or superior mesenteric vein resection and reconstruction is a safe and effective surgical procedure. However, identifying suitable revascularization materials remains challenging.

**Patient concerns:**

We report a rare case of 11-year patency following reconstruction of the ligamentum teres hepatis with vein reconstruction. The patient’s diagnosis, treatment, and postoperative follow-up are summarised and analysed.

**Diagnoses:**

The patient was diagnosed with a neuroendocrine tumor of the pancreatic head.

**Interventions:**

We conducted a pancreaticoduodenectomy with resection of a 3-cm segment at the portal superior mesenteric vein confluence.

**Outcomes:**

We reconstructed the vein using a recanalized autologous ligamentum teres hepatis (LTH) graft. No long-term anticoagulation therapy was administered. The graft remained patent throughout an 11-year postoperative follow-up, during which the patient remained tumor-free and in good general condition.

**Lessons:**

This case provides rare long-term data supporting the feasibility of using the LTH as an autologous graft for venous reconstruction. Given its accessibility, structural similarity to native veins, and potential for long-term patency, the LTH may be considered a viable substitute in complex portal venous reconstruction during pancreatic surgery.

## 1. Introduction

Pancreatoduodenectomy (PD) is the primary surgical method used to treat cholangiocarcinoma, pancreatic head cancer, and periampullary cancer. When a tumor invades the portal vein (PV) or superior mesenteric vein (SMV), combined PV or SMV resection and reconstruction is often required to achieve radical resection. The selection of repair and reconstruction materials is of paramount importance. The feasibility of the ligamentum teres hepatis (LTH) as an emerging revascularization graft for vein reconstruction in combined PD with PV resection, as well as its short-term efficacy, has been demonstrated.^[1,2]^ However, its long-term efficacy has not yet been reported. We performed partial pancreaticoduodenectomy with combined PV resection and LTH reconstruction in a patient with a pancreatic head tumor requiring intraneural division in 2012. The vascular graft remained patent at 11 years postoperatively during surveillance imaging. This study represents the longest documented follow-ups for LTH graft patency. The treatment experience is reported as follows.

## 2. Case description

A 51-year-old male with a body mass index of 22.9 kg/m² was admitted on April 9, 2012, due to progressive worsening of jaundice over a 15-day period. He had no significant medical history. On physical examination, icteric sclera and skin were observed. The abdomen was soft and non-tender, with no palpable masses. The liver and spleen were non-palpable, and there was no palpable gallbladder distension.

Laboratory investigations revealed the following: total bilirubin, 106.0 μmol/L (reference range: 3.4–20.5 μmol/L); carbohydrate antigen 19-9, 231.0 U/mL (reference range: <37 U/mL); carcinoembryonic antigen, 5.79 ng/mL (reference range: <5.0 ng/mL); alanine aminotransferase, 111.8 U/L (reference range: 7–40 U/L); alkaline phosphatase, 963.5 U/L (reference range: 40–150 U/L); and fasting blood glucose, 5.12 mmol/L (reference range: 3.9–6.1 mmol/L). Upper abdominal computed tomography (CT) revealed dilation of both intrahepatic and extrahepatic bile ducts, marked stenosis of the pancreatic segment of the common bile duct, dilatation of the pancreatic duct, and enlargement of the pancreatic head. No endoscopic ultrasound biopsy or radionuclide scan was established. Following a multidisciplinary preoperative discussion, a diagnosis of pancreatic head cancer was made. The patient underwent operation on April 12, 2012.

After tracheal intubation under general anesthesia, a midline epigastric incision was made. The tumor, approximately 4 cm × 4 cm × 3 cm in size, was located in the head of the pancreas, and was found to invade the right posterior wall of the PV and SMV confluence over a length of approximately 2 cm. Enlarged lymph nodes were noted in the surrounding area. The common bile duct was thickened, with a diameter of 2.0 cm, and the gallbladder was distended. No abnormalities were observed in the liver, gastrointestinal (GI) tract, or pelvic peritoneum.

PD combined with segmental resection of the portal superior mesenteric vein (PSMV) and reconstruction using the LTH was proposed. The patient’s LTH was excised, trimmed, and dilated to form a venous graft approximately 1.0 to 0.8 cm in diameter, which was preserved in heparinized saline. Approximately 50% of the distal stomach, duodenum, 20 cm of the proximal jejunum, the head of the pancreas, the lower portion of the bile duct, gallbladder, and the involved PSMV segment were resected. The splenic vein was ligated. A 3-cm segment of the involved vein was resected, and a 3-cm LTH graft was used for reconstruction. Vascular end-to-end anastomosis was performed, first to the SMV and then to the PV. A 5-0 Prolene suture was used for continuous suturing, with the posterior wall anastomosed first, followed by the anterior wall. Upon completion, PV blood flow was restored and confirmed to be smooth. The total PV occlusion time was 40 minutes.

**Figure 1.**
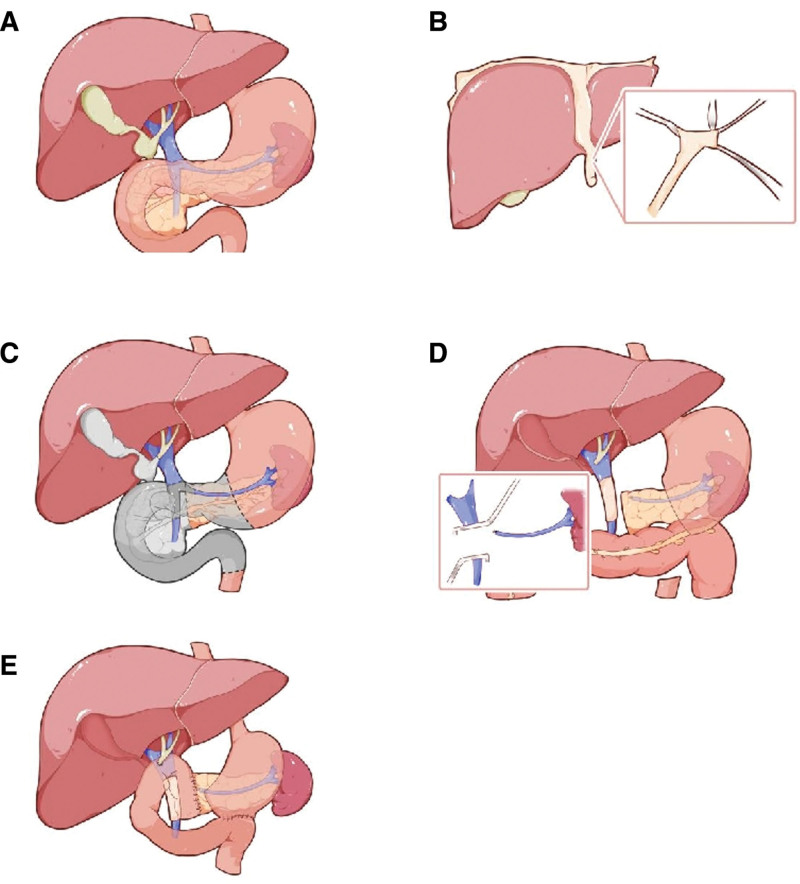
Schematic diagram of the surgical procedure. (A) Localization of the tumor. (B) Dilation and recanalization of the LTH. (C) Extent of surgical resection. (D) Resection of segment of the portal vein and replacement using recanalized LTH. (E) Reconstruction of the gastrointestinal tract. LTH = ligamentum teres hepatis.

A child’s reconstruction procedure was performed to restore GI continuity. A jejunostomy tube was inserted for enteral nutrition, and T-tubes and pancreatic duct drains were placed for external drainage of bile and pancreatic secretions. No hepatic artery injury occurred during the surgical procedure. An abdominal drain was also inserted. A schematic representation of the surgical procedure is shown in Figure 1. The operative time was 400 minutes, and intraoperative blood loss was 300 mL.

On postoperative day (POD) 1, early enteral nutrition via jejunostomy tube was initiated and supplemented with parenteral nutrition. Biliary and pancreatic secretions were collected and reinfused into the jejunostomy. Return of bowel function was noted on POD 3, with passage of flatus and stool, and the patient was gradually transitioned to an oral diet beginning on POD 7. Doppler ultrasonography performed on POD 20 confirmed patency of the PSMV complex, with no evidence of thrombus formation within the vascular graft.

**Figure 2.**
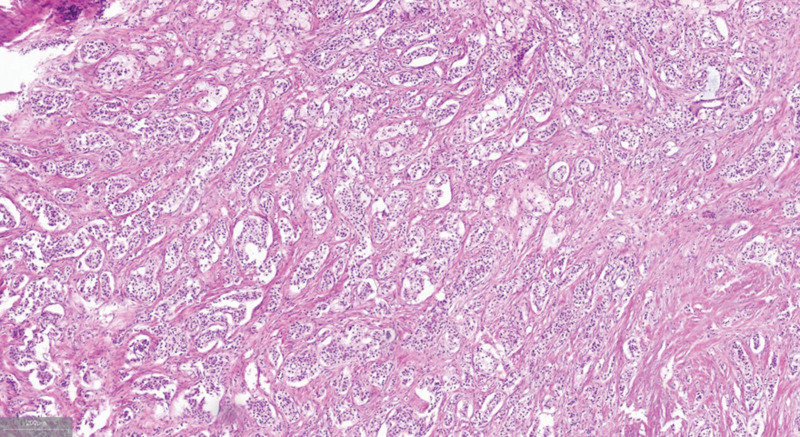
Representative pathological sections confirming a pancreatic neuroendocrine tumor with portal vein wall invasion and lymph node metastasis.

Histopathological examination confirmed a pancreatic neuroendocrine tumor with duodenal serosal invasion and PV wall involvement. Metastasis to 1 of 16 examined pancreatic lymph nodes was observed (Fig. 2).

The patient experienced no postoperative pancreatic or biliary leaks. On POD 8, abdominal and pleural effusions were identified, which resolved after percutaneous drainage. The patient’s general condition gradually improved, and drainage tubes were gradually removed. He was discharged on POD 28.

**Figure 3.**
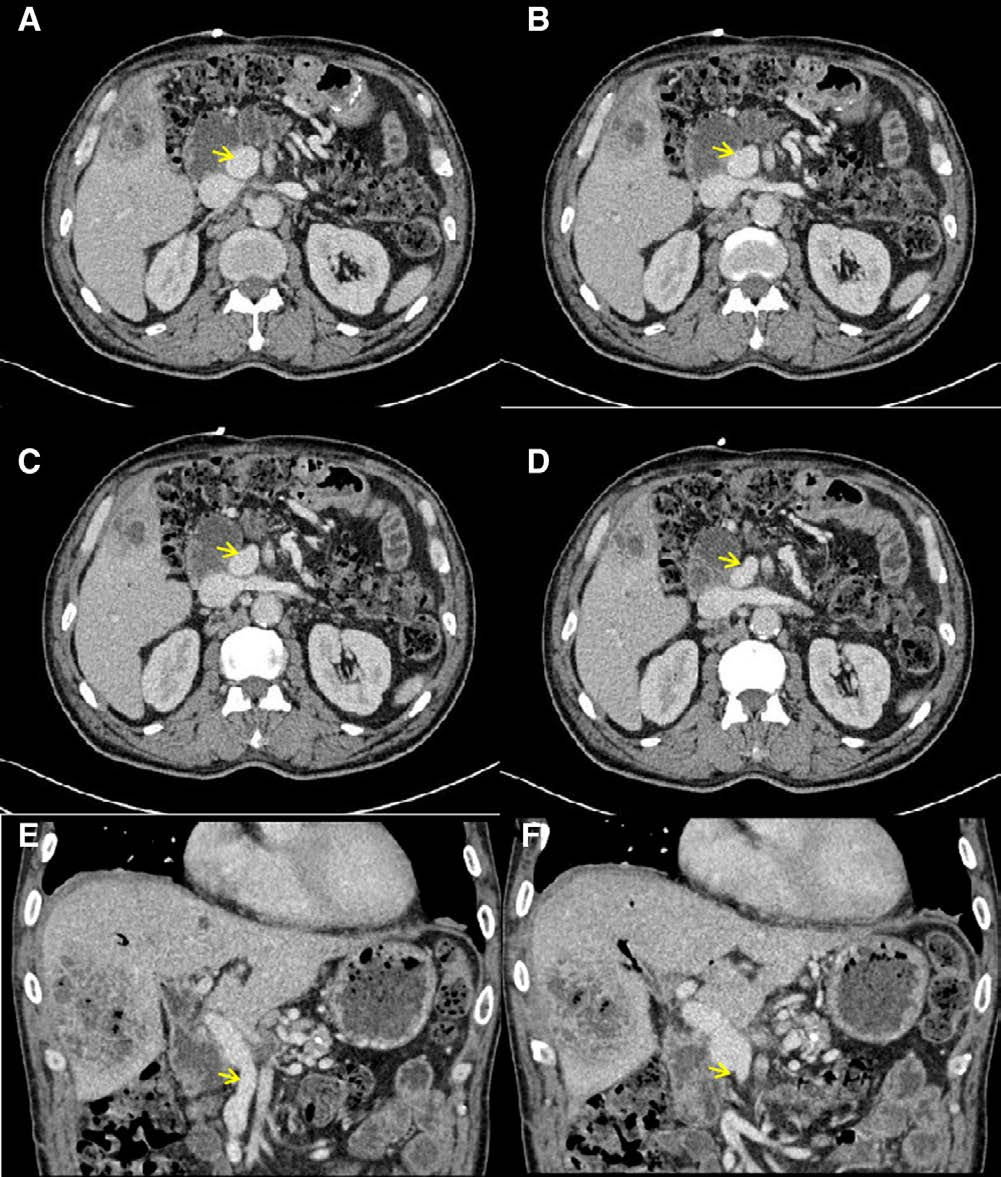
Enhanced CT performed on May 30, 2017. (A–D) Axial enhanced CT image, with the yellow arrow indicating the portal superior mesenteric vein. (E, F) Coronal enhanced CT image, with the yellow arrow indicating the ligamentum teres hepatis graft. CT = computed tomography.

**Figure 4.**
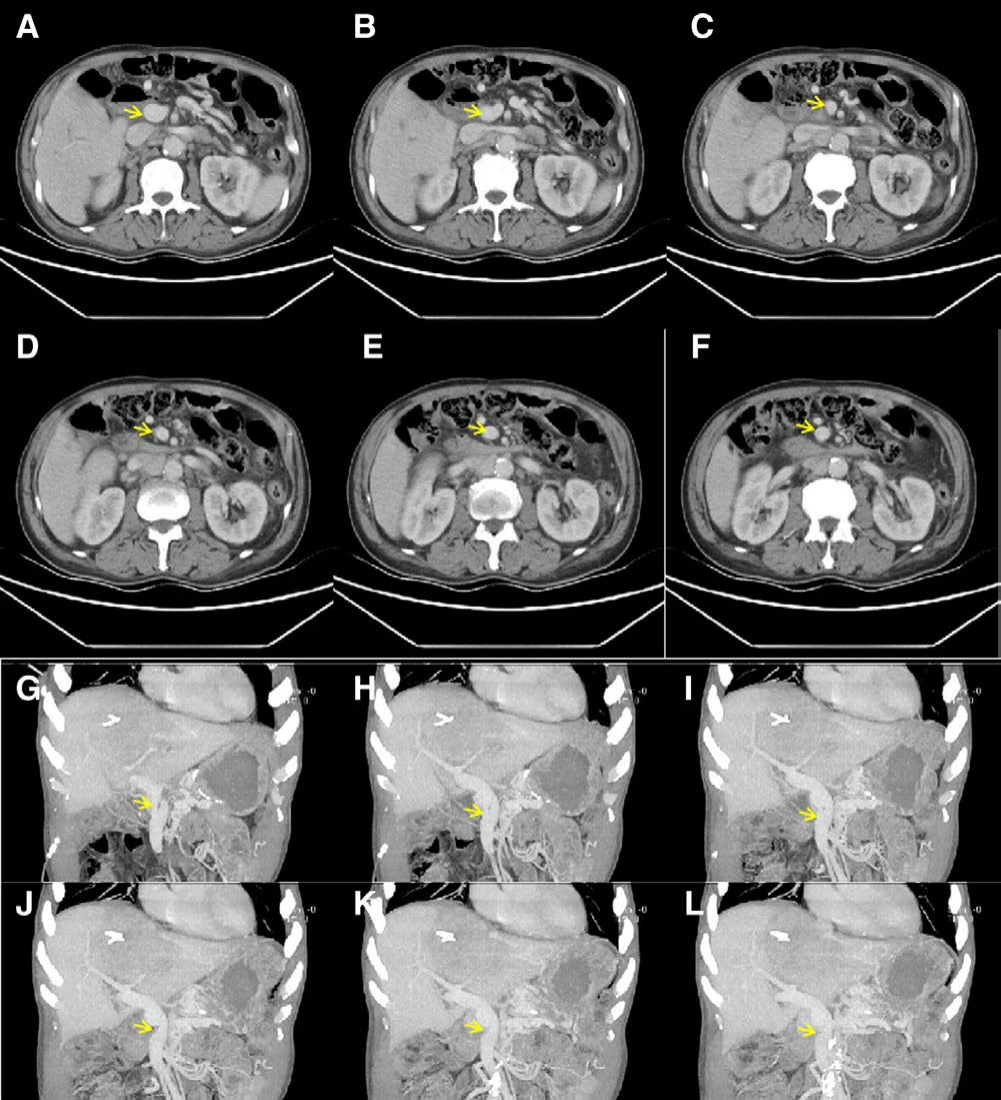
Enhanced CT performed on January 19, 2023. (A–F) Axial enhanced CT image, with the yellow arrow indicating the portal superior mesenteric vein. (G–L) Coronal enhanced CT image, with the yellow arrow indicating the ligamentum teres hepatis graft. CT = computed tomography.

The patient has been followed-up regularly for 11 years after surgery, with no evidence of tumor recurrence. He remains in good general condition and lives independently. During the follow-up period, the patient experienced bacterial liver abscesses at multiple postoperative intervals (4, 5, 6.5, and 10.8 years). The first 3 abscesses were located in the left hepatic lobe, the junctional area between the left and right hepatic lobes, and the right hepatic lobe, respectively. The fourth abscesse presented as multifocal intrahepatic lesions. All abscesses resolved completely following percutaneous catheter drainage and antibiotic therapy.The patient experienced 2 separate episodes of upper GI bleeding in the seventh and eighth years postoperatively. Endoscopic examination confirmed bleeding from gastrojejunal anastomotic ulcers, with no evidence of gastroesophageal varices. Both episodes resolved completely with medical therapy. Postoperative enhanced CT examinations were performed on March 25, 2016; May 30, 2017; September 21, 2018; and January 19, 2023, respectively. All studies consistently showed normal PV inner diameter and smooth flow through the LTH graft without thrombosis, with corresponding graft diameters measuring 1.44, 1.46, 1.52, and 1.67 cm, respectively (Figs. 3 and 4).

## 3. Discussion

Currently, the primary materials used for PV repair and reconstruction include artificial prostheses, autologous veins such as the internal jugular, femoral, iliac, gonadal, saphenous, splenic, and renal veins, as well as cryopreserved allografts.^[3]^ However, harvesting autologous vascular vessels involves additional incisions and dissection, which increases the complexity of surgery and carries a risk of complications, including injury to vital vessels.^[4-10]^ Patency rates of autologous grafts depend on multiple factors such as the graft type, harvesting method, surgical technique, and postoperative anticoagulation. For example, the left renal vein has shown patency rates of approximately 83%,^[9,10]^ whereas the saphenous and femoral veins have achieved rates of 88% and 80%, respectively, within 3 to 6 months postoperatively.^[4,5]^ Nonetheless, observational studies on long-term patency remain limited.

Although artificial vessels and allografts avoid donor site morbidity, they carry risks of immune rejection, which may lead to thrombosis or infection. Stauffer et al^[11]^ reported 33 patients who underwent PV or SMV reconstruction with prosthetic grafts from 1994 to 2009; 3 developed graft thrombosis within 30 days, with an overall patency rate of 76%. The actual patency rates at 6 and 12 months postoperatively were 77.3% and 64.3%, respectively. Meniconi et al^[3]^ reviewed PV reconstructions using refrigerated iliac or femoral vein allografts. No thrombosis or stenosis occurred, and all grafts remained patent during follow-up (median, 8 months).

The LTH, formed by the embryonic degeneration of the left umbilical vein, connects the umbilical ring to the capsule of the PV’s left branch. In 2003, we successfully employed LTH for venous reconstruction during PD. We subsequently conducted anatomical and histological studies examining the length of dilated and recirculated LTH, histological characteristics of the wall, and endothelial distribution, demonstrating that its structure closely resembles that of the PV, supporting its feasibility as a vascular substitute.^[12-14]^ In recent years, reports have emerged of using artificially recanalized LTH for reconstruction of the PV, SMV, inferior vena cava, or hepatic vein in PD and liver transplantation.^[1,15-18]^

In our center, we retrospectively analyzed 26 patients who underwent PD with PV or SMV resection using LTH grafts. Mild postoperative graft stenosis was observed in 19.23% of cases; however, all grafts remained patent, yielding a 100% patency rate.^[2]^ This suggests that LTH provides excellent short-term outcomes when used in venous reconstruction.

Long-term patency of LTH grafts remains uncertain due to the generally poor long-term survival of patients undergoing these surgeries. In the present case, a patient with a pancreatic neuroendocrine tumor maintained vascular graft patency for 11 years without anticoagulation therapy. A 3-cm segment of LTH used for PV and SMV reconstruction remained patient without thrombosis or significant changes in diameter, demonstrating the feasibility of LTH dilatation and recanalization for long-term vascular grafting. Our study revealed that the inner wall of the dilated and recanalized LTH possesses an intact, functional endothelial cell layer containing normal levels of factor VIII and CD34, which effectively prevents thrombosis.^[2,12]^ This is the primary reason why the graft remained patent in this case despite the absence of long-term postoperative anticoagulation therapy.

In this patient, the affected vessel involved the confluence of the splenic vein and SMV. The splenic vein was partially resected and ligated without reconstruction. Follow-up imaging showed mild regional varices without significant splenomegaly. The patient developed recurrent bacterial liver abscesses 4 times starting in the fourth year postoperatively, potentially related to reflux of intestinal contents into the biliary system following child’s procedure reconstruction. No hepatic artery injury was observed intraoperatively, and postoperative CT demonstrated adequate hepatic arterial perfusion, thus ruling out hepatic abscesses caused by hepatic arterial hypoperfusion. The patient experienced 2 episodes of GI bleeding postoperatively. Gastroscopy confirmed these were caused by a gastric-jejunal anastomotic ulcer. No gastric fundus esophageal varices were identified, ruling out the possibility of regional portal hypertension bleeding due to splenic vein ligation. Additionally, during the occurrence of these 2 complications, the LTH graft remained patent, and blood flow in the portal venous system was normal. Therefore, the emergence of complications showed no apparent association with the LTH graft. In conclusion, LTH is an easily accessible autologous material that avoids donor site morbidity, supports high rates of recanalization and long-term patency, and demonstrates promise as a graft option for venous reconstruction during PD with PV resection. However, this report is limited by its single-patient design, and the findings may not be generalizable to all clinical settings. Further studies involving larger cohorts, long-term follow-up, and comparative analyses are warranted to validate the efficacy and safety of LTH grafts and to establish standardized indications for their use in complex venous reconstructions. Secondly, another limitation of this study lies in the lack of standardized and predefined intervals for postoperative imaging follow-up, which may have resulted in insufficient precision in observing the morphological and functional evolution of the graft at critical postoperative time points. Despite these limitations, the long-term patency and excellent clinical outcomes demonstrated in this case undoubtedly provide valuable preliminary evidence for the application of the LTH in PV reconstruction, pointing the way for further exploration.

## Author contributions

**Conceptualization:** Zhiwei Liu, Qiang Wei.

**Data curation:** Qiang Wei.

**Formal analysis:** WenTao Zhu.

**Funding acquisition:** QiangPu Chen.

**Investigation:** WenTao Zhu.

**Methodology:** Qiang Wei, WenTao Zhu.

**Project administration:** Zhiwei Liu.

**Resources:** Xutao Lin.

**Software:** Xutao Lin.

**Supervision:** QiangPu Chen.

**Validation:** Zhiwei Liu.

**Visualization:** Zhiwei Liu.

**Writing – original draft:** Zhiwei Liu, Qiang Wei.

**Writing – review & editing:** WenTao Zhu, Xutao Lin, QiangPu Chen.
